# Genotype-Phenotype Correlation in Chinese Patients with Spinal and Bulbar Muscular Atrophy

**DOI:** 10.1371/journal.pone.0122279

**Published:** 2015-03-26

**Authors:** Wang Ni, Sheng Chen, Kai Qiao, Ning Wang, Zhi-Ying Wu

**Affiliations:** 1 Department of Neurology and Institute of Neurology, First Affiliated Hospital, Fujian Medical University, Fuzhou, China; 2 Department of Neurology and Institute of Neurology, Second Affiliated Hospital, Zhejiang University School of Medicine, Hangzhou, China; 3 Department of Neurology and Institute of Neurology, Huashan Hospital, Shanghai Medical College, Fudan University, Shanghai, China; University of Florida, UNITED STATES

## Abstract

Spinal and bulbar muscular atrophy (SBMA) is an X-linked recessive motor neuron disease characterized by slowly progressive weakness and atrophy of proximal limbs and bulbar muscles. To assess the genotype-phenotype correlation in Chinese patients, we identified 155 patients with SBMA and retrospectively examined available data from laboratory tests and neurophysiological analyses. Correlations between genotype and phenotype were analyzed. There was an inverse correlation between the length of CAG repeats and age at first muscle weakness (*p<0*.*0001*). The serum creatine kinase level showed a significant inverse correlation with disease duration and the age at examination (*p=0*.*019* and *p=0*.*004*, respectively). Unlike previous classification of motor- and sensory-dominant phenotypes, all findings of nerve conduction, except the amplitudes of median nerve compound motor action potential, were positively correlated to the length of CAG repeats. A significant decline in sensory nerve action potential amplitudes may assist differential diagnosis of SBMA.

## Introduction

Spinal and bulbar muscular atrophy (SBMA), also termed as Kennedy’s disease, is an X-linked recessive motor neuron disease characterized by slowly progressive weakness and atrophy of proximal limbs and bulbar muscles[1]. Gynecomastia and reduced fertility caused by androgen insensitivity can also be noticeable [2].

SBMA is caused by an expansion of CAG repeats within exon 1 of androgen receptor (*AR*) gene and the number of CAG repeats beyond 38 is pathogenic [3]. As a member of polyglutamine (polyQ) diseases, the age at onset (AAO) of SBMA, which was defined as the age at first muscle weakness, is correlated inversely with the number of CAG repeats mimicked to other polyQ diseases [4–6]. Nevertheless, the number of CAG repeats isn’t correlated with the severity of disease [7, 8]. Only male patients develop clinical symptoms while female homozygotes with an expanded CAG repeats don’t present clinical phenotypes [9]. High levels of circulating hormones were found to involve in disease onset in transgenic mice and *Drosophila* models [10, 11]. The major pathogenesis of SBMA was the expanded polyQ tracts aggregation in the nuclei of motor neuron [12]. Several recent studies have further demonstrated that polyQ-expanded protein aggregates into oligomers and inclusions, and the inclusions formed by the mutant protein are supposed to be protective, while the diffuse oligomers may be toxic [13, 14]. These findings partly explain the phenotypic variation in neurophysiological studies among SBMA patients [15].

Even though clinical and genetic features of SBMA have been defined, and the correlation between genotype and phenotype has been described in Japanese and American SBMA patients [7, 8], there are still remarkable phenotypic variations among different populations. In addition, only few studies had been reported in Chinese SBMA patients with small samples [16, 17]. To elucidate the genotype-phenotype correlation in the Chinese population, we analyzed a retrospective data collected from 155 Chinese SBMA patients identified by genetic testing of *AR* gene.

## Patients and Methods

### Patients

One hundred and fifty-five SBMA male patients from one hundred and fifty-four pedigrees were consecutively recruited from a Han Chinese population between December 23, 2007 and August 6, 2014. The diagnosis of SBMA was followed the 2011 EFNS guidelines for the molecular diagnosis of neurogenetic disorders [18]. Patients with muscle disorders, peripheral neuropathy or other systemic diseases which may affect the laboratory tests were excluded. This study was approved by the ethics committee of Huashan Hospital. Written informed consent was obtained from each patient.

### Genetic analyses

Genomic DNA was extracted from peripheral blood using a QIAamp DNA Blood Minikit (QIAGEN, Hilden, Germany). A pair of primers (AR1F: TCCAGAATCTGTTCCAGAGCG, AR1R: TGAAGGTTGCTGTTCCTCATCC) were used for PCR amplification and detailed PCR conditions were reported previously [19]. The specific number of CAG repeats was further verified via Sanger sequencing.

### Laboratory tests

Eighty-six patients received serum creatine kinase (CK) measurements. Thirty-two and forty-four of them further received measurements of serum aspartate aminotransferase (AST) and lactic dehydrogenase (LDH) respectively. Twenty-nine patients received blood lipid measurements (including blood total cholesterol (TC), blood low-density lipoprotein (LDL) and blood triglyceride (TG). The measurements of hormone level were performed with Roche cobas hormonal assay on Roche E170 analyzer. The values of CK, AST, LDH, lipids and hormone level were obtained before medication intervention. All the laboratory tests were performed by the clinical laboratory. Reference range of each test was reported according to the lab normative values of Huashan Hospital.

### Neurophysiological analyses

Electromyography (EMG) and nerve conduction studies (NCS) were performed in eighty-seven patients. NCS were performed using standard methodology and reported according to lab normative values [20]. Two motor nerves (right median and peroneal nerves) and three sensory nerves (right median, ulnar and sural nerves) were included in the study. Compound motor action potential (CMAP) values and sensory nerve action potential (SNAP) values were used for the statistical analyses.

### Data analyses

Statistical analyses were performed using SPSS version 13.0 (SPSS Inc., Chicago, IL). Quantitative measures for the SBMA patients were summarized with descriptive statistics, such as mean, SD, SE, range, and 95% confidence interval (CI) of mean. Pearson’s correlation coefficients were used to assess the relationship between the number of CAG repeats and other possible influencing factors. Relationships between age at genetic diagnosis and laboratory test values were also assessed by Spearman’s correlation coefficients. Analysis of covariance (ANCOVA) and Bonferroni correction were used to compare neurophysiological values between subgroups divided according to the mean number of CAG repeats. *P*-values were two-tailed and a significant level of 0.05 was used.

## Results

### Clinical features

The clinical features and genetic backgrounds of 155 SBMA patients are shown in Table 1 (Individual data in S1 Data). Only 35 out of 155 patients have ascertained family history while 110 have no family history and 10 remain undetermined. Symmetric muscle weakness seemed to be the most common presentation in our patients, while involvement of bulbar muscles, like dysarthria and dysphagia, and symptoms caused by androgen insensitivity were not rare. Also, 96% of our patients had onset weakness of limb muscle, followed by 3% of bulbar weakness (Table 2).

**Table 1:** Demographics of spinal and bulbar muscular atrophy (SBMA) patients.

	Mean ± SD (range)
**Disease milestones**	
Age at first muscle weakness	44.2 ± 10.2 (24–71)
Age at genetic diagnosis	51.0 ± 10.1 (25–74)
Mean intervals between AAO to age at genetic diagnosis	6.6 ± 5.4 (1–34)
**Genetics**	
Length of CAG repeats	48.6 ± 3.5 (42–61)
**Family history**	
Positive	35 (22.6%)
Negative	110 (71.0%)
Unknown	10 (6.4%)

**Table 2:** Symptoms and onset distribution in 155 SBMA patients.

	Number (%)
**Symptoms**	
Bulbar weakness	55 (35.5%)
Arm weakness	101 (65.2%)
Leg weakness	117 (75.5%)
Tremor	21 (13.5%)
Breast enlargement	54 (34.8%)
**Distribution of first muscle weakness**	
Bulbar	4 (2.6%)
Arm	21 (13.5%)
Leg	61 (39.4%)
Both arm & leg	67 (43.2%)
Asymptomatic CK elevation	2 (1.3%)

Clinical symptoms were collected when the patients first came to the clinic. Some patients were presenting more than one symptom. Distribution of first muscle weakness noted as “Both arm & leg” means the patients could hardly recognise the earliest onset muscle.

### Correlation between number of CAG repeats and onset age

**Fig 1:**
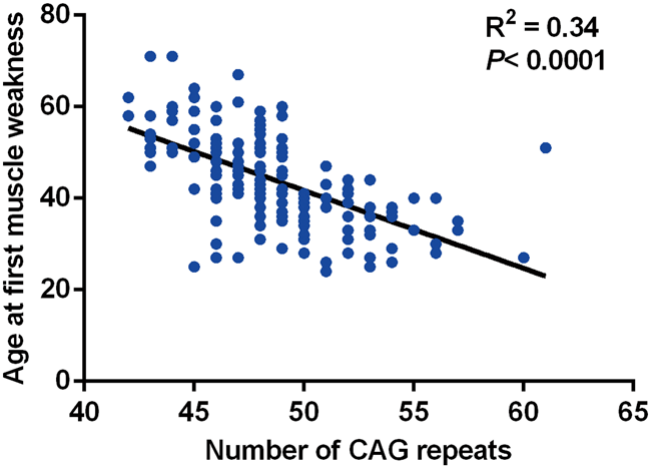
Relationship between the number of CAG repeats and the age at first muscle weakness. There is an inverse relationship between the number of CAG repeats and the age at first muscle weakness (R^2^ = 0.34, *p<0*.*0001*).

The average number of CAG repeats was 48.6 ± 3.5 (Table 1). There was an inverse correlation between the number of CAG repeats and the onset age of muscle weakness (*p<0*.*0001*, Fig. 1). The number of CAG repeats could explain approximately 34% variance of age at first muscle weakness.

### Correlation between number of CAG repeats and values of laboratory tests

The laboratory tests of patients, like serum CK, LDH, and triglycerides (TG) showed a mild to moderate abnormality. Biochemical and hormonal data are listed in Table 3 (Individual data in S1 Data). Serum CK levels were elevated in 85 out of 86 patients, particularly in the early stage of the disease. 91% (29/32) and 86% (38/44) of patients presented an elevation of serum AST and LDH levels, respectively. Hypertriglyceridemia was the major finding and 66% (19/29) of patients had a history of hypertriglyceridemia. In addition, only 22% (6/27) had high level blood total cholesterol (TC) and 12% (3/26) had elevated blood low-density lipoprotein (LDL). Half of patients (17/34) showed an elevation in blood estradiol level. Four patients were found to have low blood testosterone level accompanied by another 5 elevated (Table 3). None of these laboratory tests’ values showed a significant association with the number of CAG repeats. However, the CK level showed a significant inverse correlation with disease duration and the age at examination (*p = 0*.*019* and *p = 0*.*004*, respectively).

**Table 3:** Values of laboratory tests in spinal and bulbar muscular atrophy (SBMA) patients.

	Mean ± SE(range)[95% CI]	Reference range*	Out of reference range (%)	
			Low	High
CK (U/L) (N = 87)	1024 ± 76 (165–3193) [872,1177]	38–174	0	85 (98%)
AST (U/L) (N = 32)	51 ± 4 (29–142) [43,58]	<30	-	29 (91%)
LDH (U/L) (N = 44)	275 ± 10 (162–427) [255,296]	106–211	0	38 (86%)
TG (mmol/L) (N = 29)	2.68 ± 0.30 (0.65–7.70) [2.07,3.29]	<1.80	-	19 (66%)
TC (mmol/L) (N = 27)	5.04 ± 0.17 (3.43–6.98) [4.71,5.39]	2.80–5.90	0	6 (22%)
LDL (mmol/L) (N = 26)	2.84 ± 0.15 (1.54–4.48) [2.52,3.15]	1.30–3.70	0	3 (12%)
Estradiol (μmol/L) (N = 34)	144.5 ± 10.3 (40–301) [123.7,165.4]	28–156	0	17 (50%)
Testosterone(nmol/L) (N = 29)	19.8 ± 1.54 (8.39–40.62) [16.60,22.92]	9.90–27.80	4 (14%)	5 (17%)

*Reference ranges are reported according to lab normative values of Huashan Hospital.

### Correlation between number of CAG repeats and neurophysiological values

We performed EMG and NCS assessments in 87 SBMA patients. The average number of CAG repeats was 48.7 ± 3.8. All of them showed neurogenic motor unit action potential with amplitudes higher than 5 mV in at least 3 segments in needle EMG study. Mild fibrillations and positive sharp waves were observed frequently. We included two motor nerves (right median and peroneal nerves) and three sensory nerves (right median, ulnar and sural nerves) in the nerve conduction studies (Table 4) (Individual data in S1 Data). Only few patients showed decreased nerve conduction velocities. However, 28% of patients showed decreased amplitude of the median nerve CMAP, and 43% showed decreased amplitude of the peroneal nerve CMAP. 85%, 100% and 52% of patients developed low SNAP amplitudes in the median, ulnar and sural nerves, respectively. We then divided our patients into two groups according to the average number of CAG repeats, 49. ANCOVA indicated peroneal nerve and three sensory nerves have significant differences between subgroups except for the median nerve CMAP when the disease duration before examination was adjusted (Table 5).

**Table 4:** Analysis of nerve conduction in spinal and bulbar muscular atrophy (SBMA) patients.

	Mean ± SD (range)	Reference range	Abnormally low (%)
**Motor nerve conduction**			
Median CMAP(mV)	5.9 ± 2.6 (0.2–18.5)	≥ 4.5mV	24/86 (28%)
Median nerve conduction velocity(m/s)	55.7 ± 5.0 (40.5–65.7)	≥ 49m/s	6/85 (7%)
Peroneal CMAP(mV)	3.0 ± 1.9 (0–8.4)	≥ 2.5mV	36/83 (43%)
Peroneal nerve conduction velocity(m/s)	41.8 ± 13.1 (0–68.3)	≥ 40m/s	13/81 (16%)
**Sensory nerve conduction**			
Median SNAP(mV)	8.7 ± 6.9 (0–31.0)	≥15μV, ≥10μV over 60 years	71/84 (85%)
Median nerve conduction velocity(m/s)	56.9 ± 12.8 (0–72.2)	≥ 50m/s	9/86 (10%)
Ulnar SNAP(mV)	4.4 ± 3.4 (0–14.0)	≥15μV, ≥10μV over 60 years	82/82 (100%)
Ulnar nerve conduction velocity(m/s)	55.8 ± 12.9 (0–72.4)	≥ 50m/s	15/84 (18%)
Sural SNAP(mV)	5.8 ± 4.8 (0–23)	≥6μV, may be absent over 60 years	44/84 (52%)*
Sural nerve conduction velocity(m/s)	47.0 ± 13.7 (0–67.3)	≥ 40m/s	9/86 (10%)

*Sural SNAP may be absent in patients over 60 years, thus patients over 60 years were excluded.

**Table 5:** Neurophysiological values between subgroups divided according to the mean number of CAG repeats.

	Adjusted mean amplitudes ± SE*		***P*** values between subgroups
	CAG ≤ 49	CAG > 49	
Median CMAP (mV)	6.000 ± 0.323	5.613 ± 0.506	0.523
Peroneal CMAP (mV)	2.655 ± 0.215	3.831 ± 0.320	0.003
Median SNAP (μV)	6.723 ± 0.819	12.823 ± 1.260	< 0.001
Ulnar SNAP (μV)	3.439 ± 0.407	6.339 ± 0.615	< 0.001
Sural SNAP (μV)	4.447 ± 0.550	8.658 ± 0.872	< 0.001

*Adjusted for the disease duration of spinal and bulbar muscular atrophy (SBMA) patients.

## Discussion

SBMA is a slowly progressive neurodegenerative disease. Although an expansion of CAG repeats within exon 1 of *AR* is well-known to the cause of SBMA, the pathogenic mechanism remains unclear, and the precise natural history and clinical features in different populations have not been fully described. Here, we described the clinical and neurophysiological features, together with laboratory data, in 155 identified Chinese patients with SBMA. In our study population, the mean age at disease onset, which was defined as the time that the first muscle weakness appeared, was 44.2 years. The mean intervals between age at first muscle weakness and age at genetic diagnosis was 6.6 years, which is similar to that reported by Rhodes *et al*. and much shorter than that reported by Atsuta *et al*.[7, 8]. Unexpectedly, only 35 out of 155 SBMA patients have ascertained family history, which is far more less than that reported by Rhodes *et al*.[8]. We think it could be partly owing to the X-linked recessive inheritance, low recognition of disease and China’s One Child Policy. Limb muscle weakness is the major clinical manifestation of our patients. However, bulbar muscle weakness and mild to moderate gynecomastia seem much more valuable for disease differential diagnosis.

The mean length of CAG repeats in our patients was 48.6 ± 3.5, slightly more than the previous reports [7, 8]. The number of CAG repeats could explain 57% to 87% of disease AAO variance in other polyQ diseases [21–23]. Similar to a previous study [24], the number of CAG repeats could only explain at most 34% of disease AAO variance in our SBMA patients. We believe that even though the family genetic backgrounds play an critical role in the residual variance like that in other polyQ diseases [25], other possibilities including the loss of native function of *AR* as well as other unknown modifying factors may involve a lot.

In our study, the patients’ laboratory data, such as serum CK, AST, LDH, and TG displayed moderate abnormalities. Especially, the CK levels appeared to be declined with the disease duration and the age at examination. Besides, we found a more obvious elevation of blood TG than that of TC or LDL. Nearly two thirds of our patients had a history of hypertriglyceridemia. Excessive circulating TG may be referred to lipid metabolic disorders in mitochondria. Recent research in stably transfected cell lines of mutant *AR* gene and transgenic mice model indicated that the expression of Bax, caspase 3 and caspase 9 were up-regulated, which resulted in disturbance of mitochondrial homeostasis [26]. Moreover, decreased mtDNA copy number and increased frequency of 4977bp deletion of mtDNA (mtDNA^4977^) corresponded to the number of CAG repeats were found in the leukocytes of SBMA patients [27]. The total amount of testosterone in the blood was reported to be one of the determinant factors in disease progression by Rhodes *et al*.[8], however, the similar result was not shown in our patients. Small samples, hormone assay method bias and absence of DHT test in our study may be one of the reasons leading to research variation. However, we consider the elevation of estrogen, in some degree, may be more informative than blood testosterone level in the differential diagnosis of SBMA. In normal condition, only 0.3% of testosterone is converted into estradiol by CYP19A1 [28]. Due to the insensitivity of androgen receptors caused by mutant AR, the excessive blood testosterone reveals no biological androgenic effect and is transformed to estradiol, thus activates the estrogen receptors, which cause an elevation of the blood estradiol level [29]. We suspect that the elevated estradiol level also contributes to the disease pathogenesis.

The neurophysiological assessments revealed a more significant decrease in SNAP amplitudes than CMAP, which is similar to the recent reports [30]. This specific decrease of SNAP amplitude in SBMA patients may be informative in the differential diagnosis of motor neuron diseases.

It has been demonstrated in a previous study that the size of CAG repeats is closely linked to the clinical manifestations and neurophysiological findings in SBMA patients though the clinical phenotype has not been generally described [15]. In our subgroup analysis, we found that the decrease of SNAP amplitudes was much more severe in the subgroup with CAG repeats less than 49. This finding further confirms the hypothesis that patients with CAG repeats less than 49 could be more vulnerable to the sensory neuron or sensory axon impairment. In a previous report [15], subgroups of CMAP and SNAP amplitudes had been compared using Student’s t-test. Significant differences were reported in the subgroup of SNAP amplitudes, while no comparable differences but a tendency of decline was shown among the CMAP amplitudes [15]. This observation has led to an assumption of classification of motor- and sensory-dominant phenotypes. In contrast, ANCOVA analyses in our study revealed that once disease duration before examination had been adjusted, higher peroneal nerve CMAP amplitudes were surprisingly shown in the subgroup with CAG repeats more than 49 accompanied. Previous immunohistochemistry studies indicated that the extent of diffuse nuclear accumulation of mutant AR in the spinal anterior and posterior horn neurons might be positively correlated with the size of CAG repeats, but the analysis of patients’ SNAP by the same research group challenged their prior immunohistochemistry finding [15, 30, 31]. We believe the influence of disease duration should not be excluded, which made the process of mutant AR accumulation dynamic in both motor and sensory neurons.

In summary, we have analyzed the genotype-phenotype correlation in 155 Chinese patients with SBMA. Some identical features were found in this cohort of patients. The serum CK level showed a significant inverse correlation with disease duration and the age at examination (*p = 0*.*019* and *p = 0*.*004*, respectively). Unlike previous classification of motor- and sensory-dominant phenotypes, all findings of nerve conduction, except the amplitudes of median nerve CMAP, were positively correlated to the length of CAG repeats. A more significant decline in SNAP amplitudes than CMAP may be an important feature of SBMA patients. However, a prospective study with a larger study population is still needed to better characterize the genotype-phenotype correlation of SBMA patients.

## Supporting Information

S1 Data Individual data of descriptive statistics. Individual data of descriptive statistics were provided in the S1 Data. **Sheet1** describes the demographics of 155 spinal and bulbar muscular atrophy (SBMA) patients. **Sheet2** describes the serum CK/AST/LDH levels in 87 SBMA patients. **Sheet3** describes the blood lipid levels in 31 SBMA patients. **Sheet4** describes the serum hormone levels in 34 SBMA patients. **Sheet5** describes the nerve conduction study in 87 SBMA patients. The case numbers in each sheet may not be referred to the same participant.
(XLS)
